# Revealing Prognostic Value of Skeletal-Related Parameters in Metastatic Castration-Resistant Prostate Cancer on Overall Survival: A Systematic Review and Meta-Analysis of Randomized Controlled Trial

**DOI:** 10.3389/fonc.2020.586192

**Published:** 2020-11-19

**Authors:** Tongyu Tong, Hanqi Lei, Yupeng Guan, Xiangwei Yang, Guolong Liao, Yamei Li, Donggen Jiang, Jun Pang

**Affiliations:** Department of Urology, Kidney and Urology Center, Pelvic Floor Disorders Center, The Seventh Affiliated Hospital, Sun Yat-sen University, Shenzhen, China

**Keywords:** metastatic castration-resistant prostate cancer, skeletal-related parameters, overall survival, prognosis, meta-analysis

## Abstract

**Background:**

The skeleton is a preferred site for prostate cancer metastasis, and once metastases occur, the disease becomes incurable. Increasing evidence indicates the prognostic value of skeletal-related parameters, but remains controversial.

**Objective:**

To perform a systematic review of the existing literature on assessing the prognostic value of alkaline phosphatase (ALP), bone-specific alkaline phosphatase (BSAP), urinary N-telopeptide (uNTx), bone scan index (BSI), and Brief Pain Inventory Short Form (BPI-SF) score in castration-resistant prostate cancer (CRPC) patients with skeleton metastasis.

**Evidence Acquisition:**

PubMed, Web of Science, Cochrane Library, Medline, OVID, and Embase between 2010 and 2019 were reviewed. Key terms included randomized trials, prostate cancer, alkaline phosphatase, bone-specific alkaline phosphatase, urinary N-telopeptide, bone scan index, and Brief Pain Inventory Short Form. Data were collected, checked, and analyzed from December 2019 to March 2020. Hazard ratios (HRs) and overall survival (OS) were extracted to estimate the relationship between the above parameters and OS in patients with metastatic prostate cancer (mPCa).

**Evidence Synthesis:**

A total of 1,055 studies were identified *via* initial screening, including 1,032 from database research and 23 from other sources. After deduplication, 164 records were further excluded according to titles and abstracts. The remaining 36 potential articles were carefully screened. In the end, 15 eligible studies syntheses, which were published between 2010 and 2019, comprised data for a total of 11,378 patients, whose mean age ranged from 66 to 72 years. The sample size ranged from 82 to 1,901 patients. And the median follow-up time ranged from 24 to 55 months. Based on 15 randomized controlled trials published between 2010 and 2019, higher ALP levels (HR = 1.60, 95% CI: 1.38–1.87 *P* < 0.001), higher BSAP levels (HR = 1.31, 95% CI: 1.11–1.54 *P* = 0.001), higher uNTx levels (HR = 1.40, 95% CI: 1.29–1.52 *P* < 0.001), BSI progression (HR = 1.18, 95% CI: 1.08–1.29 *P* < 0.001), and higher BPI-SF score (HR = 1.47, 95% CI: 1.35–1.61 *P* < 0.001) had an association with inferior OS.

**Conclusions:**

Higher levels of ALP/BSAP and uNTx, a higher BPI-SF score, and progression of BSI predict inferior OS in patients with mCRPC. More randomized control trials are needed to investigate the promising value of these parameters.

## Background

Prostate cancer (PCa) is the second most commonly diagnosed cancer worldwide, and the second leading cause of cancer-associated mortality in Western countries (1). As of 2020, the United States alone reported an estimated 190,000 new PCa cases, resulting in quality of life deprivation for about 30,000 patients (1). PCa results in mortality when it metastasizes to other organs (2), and presents a disseminated status initially as metastatic hormone-sensitive prostate cancer (mHSPC) or terminal metastasis after androgen deprivation therapy (ADT) as metastatic castration-resistant prostate cancer (mCRPC).

The skeleton is the most preferential metastatic site in PCa (2), and skeleton metastasis is the main cause of mortality, impacting 65%–75% of patients (3). Metastatic PCa (mPCa) disrupts the structural integrity of the skeleton and induces resistance to conventional treatments (4). Progressive therapies such as sipuleucel T, abiraterone, enzalutamide, docetaxel, cabazitaxel, and radium-223 demonstrate limited survival benefit (5, 6). Skeletal metastasis generates skeletal-related events (SREs) including hypercalcemia, intractable pain, pathological fracture, and nerve compression syndrome, resulting in severe threat and impact on patients’ quality of life (7, 8). Studies have validated the relationship between SREs and worse OS (9–11). Therefore, the specific value for evaluating skeletal metastasis is the ability to assess disease aggressiveness and prognosis.

Previous predictive parameters of prognosis for patients with PCa including albumin (12), neutrophil-to-lymphocyte ratio (NLR), platelet-to-lymphocyte ratio (PLR) (13), body mass index (14), and circulating tumor cells (15) are indicated to be of value; however, the disadvantages are obvious. Hence, superior prognostic makers are urgently needed to tackle the challenge. In this study, we focused on five valuable but controversial prognostic parameters: alkaline phosphatase (ALP), serum bone sialoprotein (BSP), urinary N-telopeptide (uNTx), bone scan index (BSI), and brief Pain Inventory Short Form (BPI-SF) score in mCRPC patients, through biochemical index, radiological features, and physical pain with bone eroding. We synthesized the relevant studies published and presented our analysis *via* meta-analysis to evaluate the prognostic value of these skeletal-related parameters.

## Evidence Acquisition

Prior to conducting this systematic review, two authors (TT and HL) established the selection criteria and research protocol. Thereafter, the protocol was discussed with all the co-authors for approval.

### Search Strategy

We conducted a systematic review in accordance with the Preferred Reporting Items for Systematic Reviews and Meta-Analyses (PRISMA) statement (16) and the Cochrane Handbook for Systematic Reviews of Interventions (17). Medline, Embase, Cochrane Library, Scopus, and ISI Web of Knowledge were searched between 2010 (publication date relative to the overall survival of metastatic prostate cancer) and 1 April 2020 for full-length articles published in English.

The published *a priori* protocol includes the search strategy. Three authors (TT, HL, and YG) participated in the literatures search, and the process was carried out by adapting the search strategy according to the different research engines. The following keywords and medical subject headings were used as search terms: (randomized OR randomised) AND (prostatic neoplasms OR prostate cancer) AND (alkaline phosphatase OR bone-specific alkaline phosphatase OR urinary N-telopeptide OR bone scan index OR Brief Pain Inventory Short-Form OR “ALP” OR “BSAP” OR “uNTx” OR “BSI” OR “BPI-SF”) AND (prognosis OR overall survival OR “OS”). The search string used within the PubMed (Medline) engine is specified in the supplementary material (Search Strategy).

Following deduplication, two review authors (JP and DJ) independently screened the titles and abstracts for eligibility. The full-text articles were retrieved and scrutinized independently by two review authors. For any incompletely reported data, study authors were contacted. Disagreements were resolved by discussion or by consulting a third author (XY, GL, or YL).

### Types of Study Design Included and Excluded

All randomized controlled trials (RCTs), quasi-RCTs (QRCTs), and prospective comparative studies were included. Other studies, such as noncomparative studies, retrospective studies, case series, and conference abstracts, were excluded. After deduplication, observational studies, editorials, commentaries, review articles, and those not subject to peer review (i.e., data from vital statistics and dissertations or theses) were excluded.

### Types of Participants Included

Adult male patients diagnosed with CRPC (castrate serum testosterone <50 ng/dL or 1.7 nmol/L plus either biochemical progression or radiological progression; definition in EAU guideline) and skeletal metastasis *via* pathological or imagological evidence were included. Patients with mHSPC, visceral metastasis, other types of cancer, or skeletal metastasis originating from other cancers were excluded.

### Types of Prognostic Values and Outcome Measures Included

The prognostic values of ALP, BSAP, uNTx, BSI, and BPI-SF were evaluated. Our primary outcome was OS, defined as date from randomization to death for any reason. The hazard ratio (HR) with 95% confidence interval (CI) for OS was stated, measured at any time point up to 1 month postoperatively, using any modality.

### Assessment of Bias Risks of Bias

The risk of bias for RCTs was assessed in accordance with Cochrane guidance (17). Additional domains were used to assess confounders in nonrandomized studies (NRSs): a pragmatic approach informed by methodological literature (18). We assessed whether each prognostic confounder was considered, whether the confounder was balanced between the intervention and the control group, and whether, if necessary, the confounder was controlled for in the analysis.

### Quality of Evidence Assessment

The Grading of Recommendations Assessment, Development, and Evaluation (GRADE) tool was used to assess the quality of evidence (QoE) for critical and important outcomes for decision making, including assessment of study design, risk of bias, directness, consistency, and precision (19).

### Data Analysis

We report data at available time points and report *P* values when available, or if unavailable, we calculated these using STATA. We conducted an intention-to-treat analysis, if data were available; otherwise, an available case analysis was performed. We did not impute missing data. In the case of incompletely reported data, we contacted the authors, but unfortunately, they didn’t respond.

For dichotomous outcomes, we report HRs and 95% CIs in forest plots: odds risks are less robust when data include 100% (20, 21). Where deemed clinically appropriate, a meta-analysis was performed using a random-effect model due to heterogeneity in study design, intervention schedule, outcome definition, and time point or modality of measurement. For continuous outcomes, we report the mean difference (MD) with standard deviation and/or range and corresponding 95% CIs and *p* values, where available.

### Statistical Analysis

Meta-analysis of the prognostic value of skeletal-related parameters were conducted using STATA software version 15.0 (Stata Corporation, College Station, Texas). HRs with 95% CI from all eligible studies were pooled *via* a meta-analysis to access the strength of skeletal-related parameters to survival endpoints. The *I* ^2^ statistic and Cochran’s Q test were used to evaluate the heterogeneity of the selected studies. The fixed-effects model was adopted in the absence of heterogeneity (*p*_heterogeneity_ > 0.1 and *I* ^2^ < 50%), because Cochran’s Q test is poorly equipped to detect heterogeneity; otherwise, the random effect model was used. Egger’s test with funnel plots was used to measure publication bias. The *P* value >0.05 indicated negligible potential publication bias. To test the reliability of the results, a sensitivity analysis was conducted by removing each single study in turn.

## Evidence Synthesis

### Quantity of Evidence Identified

**Figure 1:**
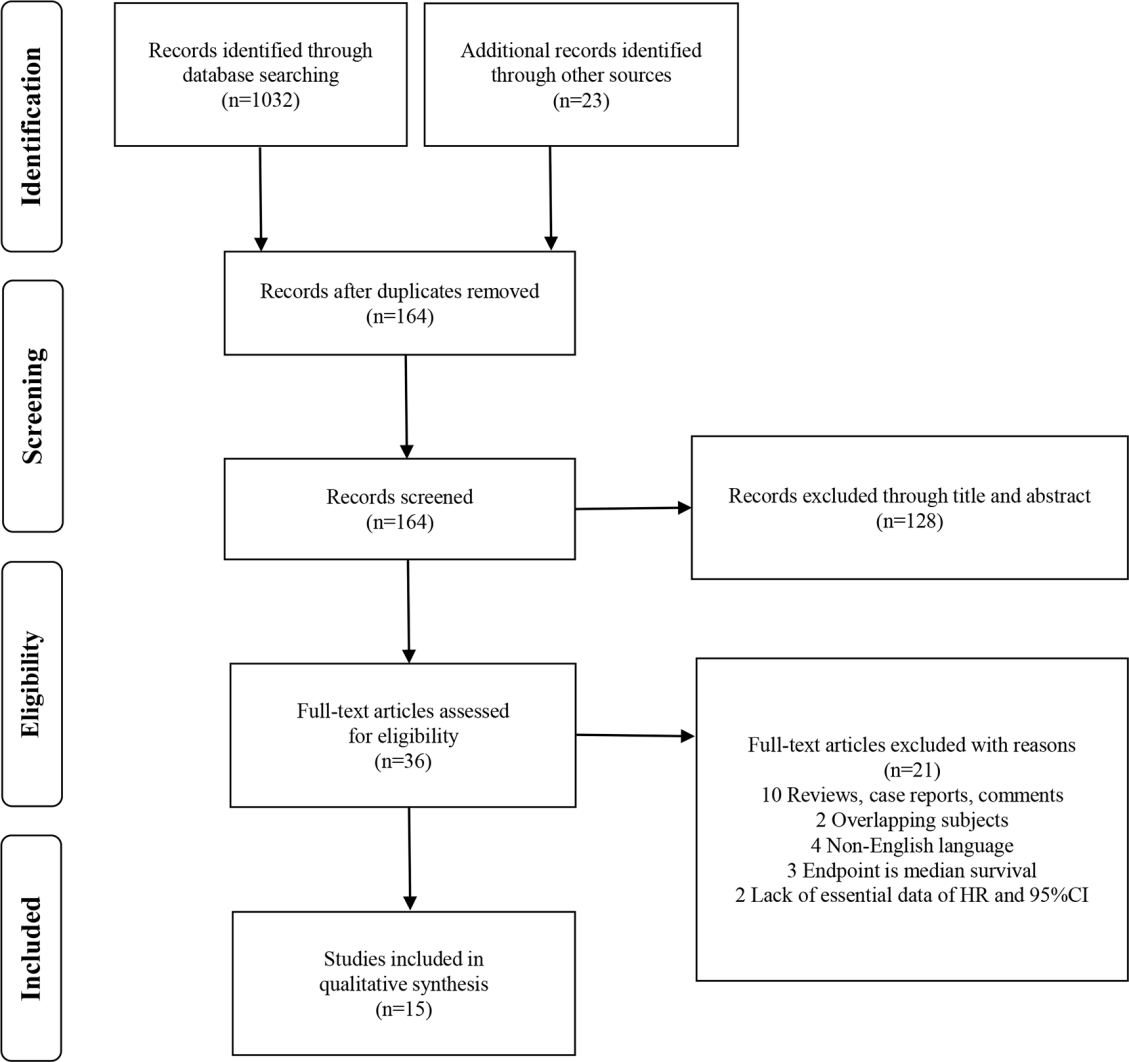
Flow chart of study selection and literature search.

The study selection process is shown as a PRISMA flow diagram (**Figure 1**). A total of 1,055 studies were identified *via* initial screening, including 1,032 from database research and 23 from other sources. After deduplication, 164 records were further excluded according to titles and abstracts. The remaining 36 potential articles were carefully screened, and 10 were ruled out for being reviews, case reports, and comments; 2 for being overlapping subjects; 4 for being non-English articles; 3 for being median survival as endpoint; and 2 for lacking essential survival data.

### Study Selection and Characteristics

Baseline characteristics of the eligible studies are presented in **Table 1** (more details are provided in **Tables S1–S4**). Finally, 15 eligible study (12, 21–34) syntheses, which were published between 2010 and 2019, comprised data for a total of 11,378 patients, whose mean age ranged from 66 to 72 years. Most studies were from America and Europe. The sample size ranged from 82 to 1,901 patients. And the median follow-up time ranged from 24 to 55 months. If there were multiple parameters in the same article, we considered them as different studies. A total of nine RCTs (12, 22, 25, 26, 28–32) had available data for analyzing the prognostic value of ALP on OS, while two RCTs (21, 28) of BSAP on OS, four RCTs (21, 23, 24, 28) of uNTx on OS, three RCTs (26, 33, 34) of BSI on OS, and four RCTs (27, 28, 30, 32) of BPI-SF score on OS. All enrolled studies were considered to be of high quality; the summary of risk of bias assessment is presented in **Figure S1**. Two of the studies (30, 34) retrospectively analyzed their own previous randomized controlled trials and we recognized that these two studies were of good quality according to the original article and the trial register numbers they published.

**Table 1:** Baseline characteristics of included studies.

Study and published year	Date	Nation/Region	Intervention	Study design	Sample size	Mean Age (range)/y	Parameter	Follow-up (range)/mo	Clinical trial
Chi et al. (22)	Sep 2005–Dec 2006	Canada/America	Docetaxel + prednisone vs docetaxel + OGX-011	Open label	82	Test: 69 (54–84) Control: 69 (49–87)	ALP	35	NCT00258388
Rajpar et al. (23)	2004–2007	France/Europe	Denosumab vs zoledronic acid	Open label	94	66(46–88)	uNTx	30 (0–34)	AMG 162 114
Araujo et al. (24)	Oct 2008–Apr 2011	USA/America	Docetaxel + dasatinib vs docetaxel + placebo	Double blind	1522	Test: 69 (45–92) Control: 68 (40–90)	uNTx	19 (11.2–25.1)	NCT00744497
Schellhammer et al. (25)	Jul 2003–Jan 2009	USA/America	Sipuleucel-T vs placebo	Open label	512	71	ALP	28	NCT00065442
Lara et al. (21)	Aug 2006–Feb 2016	USA/America	Docetaxel + prednisone + atrasentan vs Docetaxel + prednisone + placebo	Double blind	788	Test: 69 (41–92) Control: 69 (43–88)	BSAP, uNTx	48	NCT00134056
Armstrong et al. (26)	Dec 2007–Jun 2009	USA/America	Tasquinimod vs placebo	Double blind	201	Test: 72 (67–75) Control: 72 (65–78)	ALP, BSI	36	NCT00560482
Halabi et al. (12)	Apr 2005–Mar 2010	USA/America	Docetaxel + prednisone + placebo vs docetaxel + prednisone + bevacizumab	Double blind	1050	Test: 68.8 (63.0–74.4) Control: 69.3 (62.4–75.6)	ALP	48	CALGB-90401 (NCT00110214)
Goldkorn et al. (27)	Aug 2006–Feb 2016	USA/America	Docetaxel + prednisone + atrasentan vs docetaxel + prednisone + placebo	Double blind	212	Test: 69 (40–92) Control: 69 (43–89)	BPI-SF score	24	SWOG 0421 (NCT00134056)
Fizazi et al. (28)	May 2006–Oct 2009	France/Europe	Denosumab vs zoledronic acid	Double blind	1901	Test: 70.5 (61.5-79.2) Control: 71 (62.6–79.4)	ALP, BSAP, uNTx, BPI-SF score	30	NCT00321620
Soest et al. (29)	Dec 2011–May 2014	The Netherlands/Europe	Cabazitaxel + prednisone + budesonide vs cabazitaxel + prednisone	Open label	114	Test: 72 (67–75) Control: 72 (65–83)	ALP	24	CABARESC trial
Chi et al. (30)	May 2008–Aug 2010	USA/America	Abiraterone + prednisone vs prednisone	Double blind	762	Test: 69.1 (60.7–77.5) Control: 68.9 (60.3–77.5)	ALP, BPI-SF score	55	NCT00638690
Sonpavde et al. (31)	Aug 2006–Feb 2016	USA/America	Docetaxel + prednisone + atrasentan vs docetaxel + prednisone + placebo	Double blind	365	Test: 69 (40–92) Control: 69 (43–89)	ALP	30	SWOG 0421 (NCT00134056)
Miller et al. (32)	Apr 2009–Mar 2014	Germany/Europe	Abiraterone acetate + prednisone vs placebo + prednisone	Double blind	1088	Test: 70.5 (61.7–79.3) Control: 70.1 (61.4–78.8)	ALP, BPI-SF score	49.2	COU-AA-302 (NCT00887198)
Armstrong et al. (33)	Mar 2011–Aug 2015	USA/America	Tasquinimod vs placebo	Double blind	1245	70.6	BSI	42	NCT01234311
Reza et al. (34)	NA	Sweden/Europe	Zoledronic acid vs standard-of-care	Open label	1433	67	BSI	48	(ZEUS)/SPCG11

NA, not available; mHRPC, metastatic hormone-refractory prostate cancer; mCRPC, metastatic castration-resistant prostate cancer; mNCPC, metastatic non-castrate prostate cancer; ALP, alkaline phosphatase; BSAP, bone-specific alkaline phosphatase; uNTx, urinary N-telopeptide; BPI-SF, Brief Pain Inventory-Short Form score; BSI, Bone Scan Index.

### Results of Evidence Synthesis

#### Prognostic Value of ALP and BSAP for OS in mCRPC

**Figure 2:**
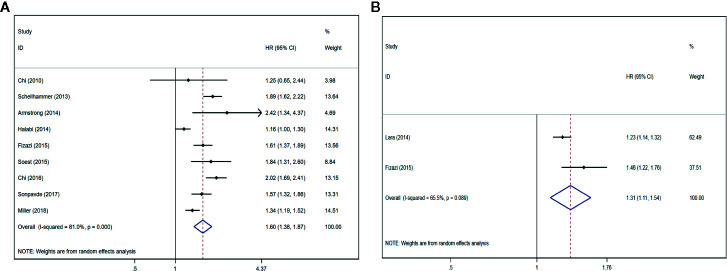
Forest plots of pooled ALP/BSAP for OS in patients with mPCa. **(A)** ALP, **(B)** BSAP. Abbreviations: HR, hazard ratio; CI, confidence interval; OS, overall survival; mPCa, metastatic prostate cancer; ALP, alkaline phosphatase; BSAP, bone-specific alkaline phosphatase.

Nine studies (12, 22, 25, 26, 28–32) described that the serum ALP content had an association with inferiorOS (HR = 1.60, 95% CI: 1.38–1.87 *P* < 0.001), with significant heterogeneity (*I* ^2^ = 81.0%, *P*_heterogeneity_ < 0.001). Forest plots (**Figure 2A**) show the pooled result. Two studies (21, 28) described that the serum BSAP content had an association with inferior OS (HR = 1.31, 95% CI: 1.11–1.54 *P* = 0.001), with significant heterogeneity (*I* ^2^ = 65.5%, *P*_heterogeneity_ = 0.089). Forest plots (**Figure 2B**) show the pooled result.

As shown in **Table 2** and **Figure S2**, to explore the potential sources of heterogeneity, a subgroup analysis and meta-regression were performed using sample size, region, study design, and serum level of ALP.

**Table 2:** Summary of the subgroup analysis results of ALP on OS.

Subgroup	Participants	Number of studies	Meta-regression *P* value	Overall survival		*I* ^2^(%)	*P*_heterogeneity_
				HR (95% Cl)	*P* value		
**Overall**	6084	9		1.60(1.38–1.87)	<0.001	81.0	<0.001
**Sample size**			0.558				
<500	771	4		1.64(1.42–1.90)	<0.001	0	0.393
500–1000	1274	2		1.95(1.73–2.19)	<0.001	0	0.559
>1000	4039	3		1.35(1.14–1.60)	<0.001	78.7	0.009
**Region**			0.988				
America	2981	6		1.65(1.30–2.09)	<0.001	86.1	<0.001
Europe	3103	3		1.52(1.28–1.80)	<0.001	59.8	0.083
**Study design**			0.996				
Open label	708	3		1.85(1.61–2.13)	<0.001	0	0.487
Double blinded	5376	6		1.55(1.30–1.85)	<0.001	84.1	<0.001
**Serum level of ALP**			0.789				
Elevated	2663	2		1.80(1.43–2.25)	<0.001	71.5	0.061
Baseline	3421	7		1.54(1.29–1.84)	<0.001	79.6	<0.001

ALP, alkaline phosphatase; OS, overall survival; HR, hazard ratio; CI, confidence interval.

#### Prognostic Value of uNTx for OS in mCRPC

**Figure 3:**
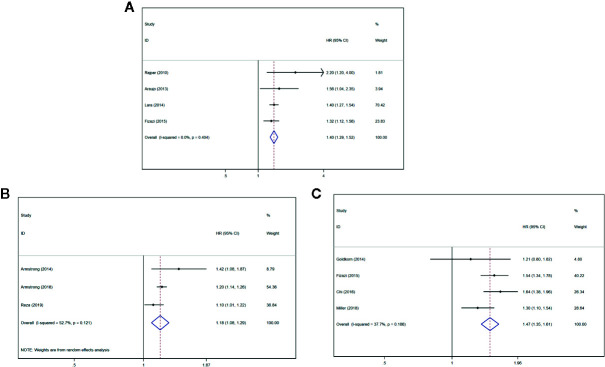
Forest plots of pooled uNTx, BSI, and BPI-SF score for OS in patients with mPCa. **(A)** uNTx, **(B)** BSI, **(C)** BPI-SF score. Abbreviations: HR, hazard ratio; CI, confidence interval; OS overall survival; mPCa, metastatic prostate cancer; uNTx, urinary N-telopeptide; BSI, Bone Scan Index; BPI-SF, Brief Pain Inventory-Short Form score.

Four studies (21, 23, 24, 28) described that the serum uNTx content had an association with inferior OS (HR = 1.40, 95% CI: 1.29–1.52 *P* < 0.001), with slight heterogeneity (*I* ^2^ = 0%, *P*_heterogeneity_ = 0.404). Forest plots (**Figure 3A**) show the pooled result.

#### Prognostic Value of BSI for OS in mCRPC

Three studies (26, 33, 34) described that the BSI progression had an association with inferior OS (HR = 1.18, 95% CI: 1.08–1.29 *P* < 0.001), with significant heterogeneity (*I* ^2^ = 52.7%, *P*_heterogeneity_ = 0.121). Forest plots (**Figure 3B**) show the pooled result.

#### Prognostic Value of BPI-SF Score for OS in mCRPC

Four studies (27, 28, 30, 32) described that the BPI-SF score had an association with inferior OS (HR = 1.47, 95% CI: 1.35–1.61 *P* < 0.001), with significant heterogeneity (*I* ^2^ = 37.7%, *P*_heterogeneity_ = 0.186). Forest plots (**Figure 3C**) show the pooled result.

### Sensitivity Analysis and Assessment of Risk of Bias

In order to evaluate the impact of a single study on the overall meta-analysis, a sensitivity analysis was performed by omitting each eligible study at a time. When each study was sequentially excluded, the results of the analysis were not significantly affected. The sensitivity analysis results demonstrated that the pooled HRs for OS did not significantly change, suggesting the robustness of the results. The details are shown in **Figure S3**.

Egger’s test was performed to assess publication bias. A study was considered to have significant publication bias when *P* < 0.05. Serum ALP level (*P* = 0.308) assessed the OS of patients with mPCa, uNTx (*P* = 0.286), BSI (*P* = 0.876), and BPI-SF score (*P* = 0.392). Egger’s test of serum BSAP level on OS could not be analyzed because of insufficient data. Publication bias analysis was not indicated in these articles (**Figure S4**).

## Discussion

As the disease progresses, PCa cells fall from their primary location, invade blood vessels, and disperse widely with blood or lymphatic circulation in the body. These PCa cells show an exquisite tropism into skeletal tissue (35). These malignant cells invade and eventually proliferate in the axial skeleton, such as the ribs, pelvis, and spine, where red marrow is most abundant (36). According to the aforementioned theories, we are interested in the prognostic value of skeletal-related parameters to assess the overall survival in patients with mCRPC.

### ALP and BSAP

In serum, variations of ALP level have long been used as an indicator of skeletal turnover and osteoblastic activity as well as indicative of the extent of bone-metastatic disease from PCa (37–39). BSAP and liver tissue-nonspecific ALP are the most abundant isoforms, comprising more than 90% of total serum ALP activity (40). BSAP is responsible for the propagation of tissue mineralization and is expressed in chondrocytes and other mineralization competent cells besides osteoblasts (41). ALP expressed by bone metastases from PCa might support osteomimicry, the ability of tumor cells that preferentially metastasize to the bone to express a genetic profile similar to the resident cells, which may allow them to have more favorable survival in the bone microenvironment during metastatic colonization (42, 43). The prognostic value of the serum ALP/BSAP level for OS in patients with mCRPC has been confirmed in multivariate analyses and multiple prognostic models independent of therapy selection (28). On account of the superiority of serum ALP-level measurement and its affordability and accessibility, ALP should be routinely monitored in patients with mCRPC besides PSA.

Our meta-analysis yielded the same result as previous research that BSAP and ALP were related to poor overall survival in patients with mPCa. We analyzed the relationship between serum level of BSAP and OS, and found that patients with a higher index had a shorter overall survival time. Only two RCTs revealed the relationship as insufficient, and more experiments are required to validate the prognostic ability of BSAP in mCRPC.

In terms of ALP, however, there was extensive heterogeneity, which would have affected the results of this meta-analysis. The sensitivity analysis demonstrated that the pooled HRs for OS did not significantly change, suggesting the robustness of the results. The results of the subgroup showed that heterogeneity was not significantly diminished besides the sample size subgroup, which means in the currently available studies, sample size had a significant effect on heterogeneity. It is suggested that consensual research is scarce and that heterogeneity may have been caused by the following factors: (a) the diversity of participant sources and interventions result in clinical heterogeneity; (b) the diversity of blinding design and sample size result in methodological heterogeneity; and (c) individual manners and subjective assessment method are used to evaluate levels of these parameters and counting effect sizes artificially result in statistical heterogeneity.

### uNTx

uNTx is a marker of bone resorption from N-telopeptides of type 1 collagen, forming 90% of organic bone matrix and cross-linked at N and C terminal ends of the molecules to form the basic fabric and tensile strength of the bone tissue (44). The uNTx sequence is generated by osteoclastic activity and proteolysis, presented as sensitive and as a specific marker to reflect activity in the bone stromal compartment (45). The enzyme-linked immunosorbent assay for the uNTx is a reliable and objective measurement. An association exists between elevated uNTx and clinical outcome, as skeletal-relevant events and inferior OS in patients with bone metastases have indicated (23, 46, 47). Here, we further demonstrate that high serum levels of uNTx is related to inferior OS in patients with mPCa, which is consistent with the results of previous articles.

### BPI-SF

In metastatic prostate cancer, skeletal involvement is a major cause of morbidity and decreased quality of life, as replacement of hematopoietic tissues by cancer cells leads to pain, bone marrow insufficiency, fractures, and spinal cord compression, and pain has been shown to predict overall survival and other clinical outcomes in patients with mCRPC (48, 49). One of the tools frequently used in pain assessment is the Wisconsin Brief Pain Inventory and its shortened version, the BPI-SF (50). The BPI-SF is a validated nine-item questionnaire and is used to assess the severity of pain. Lower scores represent lower levels of pain intensity or less interference of pain with daily activities (51). The BPI-SF item is categorized into mildly symptomatic pain, moderate pain, and severe pain based on scores of 0–4, 5–6, and 7–10, respectively. However, a large sample size RCT with 1,401 participants (52) showed there was no significant difference for BPI-SF item 3 or pain interference. In our study, we found that higher BPI-SF scores for patients with mCRPC was positively related to poor OS.

The construct validity was established through factor analysis and extraction of two factors: pain interference (factor 1) and pain severity (factor 2), and in case of reliability, α-Cronbach coefficients were high for both factors (53). A prognostic index model developed in a previous analysis identified a higher BPI-SF score as a risk factor for worse prognosis in this patient population (54). As a supplemental convenient predictive tool, BPI-SF score enables clinicians and patients themselves to assess pain severity and pain intervention, and to monitor the effect of pain management.

### BSI

Bone scintigraphy is widely used for accessing metastatic burden; however, lesions appearing in a bone scan are nonspecific and are not a direct measure of disease (26, 55). In fact, image lesions could not only be caused by metastatic disease but also by degenerative disease or fractures or healing osteoblastic reactions, which may result in misjudgment (26). BSI is a time-honored quantitative assessment of bone scan data, and represents the total tumor burden as the fraction of all skeleton weight, initially reported as an imaging biomarker for bone metastatic PCa (56). Nonetheless, time-consuming processes limited the clinical application of BSI. Here, we found that progression of BSI was positively correlated with inferior OS. Therefore, BSI shows great potential in avoiding the inaccurate subjective evaluation of and predicting mPCa prognosis.

Automated BSI (aBSI) is recognized as a further useful parameter based on BSI. Generally, aBSI dramatically reduces the calculating time from 30 minutes to 5 seconds per scan and increases the estimated sensitivity of BSI (33). It is reported that the aBSI analysis could increase the accuracy of risk stratification in these patients before the start of treatment (57) and increase the accuracy in outcome prediction during treatment (58). Thus, an evaluation system based on BSI demonstrated great potential in predicting mPCa prognosis, with the characteristics of high reproducibility and rapid processing time.

### Implications for Clinical Practice and Further Research

In summary, owing to the fact that development of bone metastases from PCa is a complex result of interaction between prostate cancer cells, osteoclasts, and osteoblasts, the prognostic value of skeletal-related parameters should be given more attention. However, few previous meta-analyses have integrated bone-related parameters to assess the prognosis, and they only analyzed unilateral parameters. This research includes more updated studies that could provide more reliable multivariate analysis adjusting HRs. Further research is required in investigating the effectiveness and clinical utility of the skeletal-related parameters assessment in patient health management to develop a more accurate and less variable method for clinical use.

### Limitations

Several limitations of this study should be acknowledged. First, although sensitivity analysis supported the stability of our results, heterogeneity was found among ALP studies and subgroup analyses as well as meta regression, thus the findings should be cautiously interpreted. Second, because of the dearth in relevant research describing the prognostic value of BSAP, we could not acquire robust conclusions. Third, examining published articles only in English may exclude studies with negative results published in other languages. Fourth, ALP, BSAP, and uNTx derived from peripheral blood or urine were easily affected by patients’ elementary conditions such as age, occupation, hepatopathy, rickets, and specific medications. Finally, many articles had positive results, as negative results are much more difficult to publish, leading to publication bias. BSI can be caused by degenerative disease or fractures, and small-size nidus and minor changes may be neglected, which results in misdiagnosis and missed diagnosis.

## Conclusions

This meta-analysis supports the prognostic value of skeletal-related parameters in mCRPC that a higher level of ALP/BSAP and uNTx, and higher BPI-SF score, and progression of BSI predict inferior OS. However, these results must be interpreted with caution because of the observed between-trial heterogeneity. More randomized controlled trials and large sample size trials are called for to confirm the potentially profound values of hematologic parameters.

## Data Availability Statement

All datasets presented in this study are included in the article/**Supplementary Material**.

## Author Contributions 

Conceptualization: TT and HL. Methodology: HL and YG. Software: TT and YG. Validation: XY, GL, and DJ. Formal Analysis: TT and HL. Investigation: TT. Resources: YG. Data Curation: TT. Writing—Original Draft Preparation: TT and HL. Writing—Review and Editing: YL and JP. Visualization: JP. Supervision: DJ. Project Administration: JP. Funding Acquisition: JP. All authors contributed to the article and approved the submitted version.

## Funding

The present study was funded by the National Natural Science Foundation of China (81772754), Major Basic Research and Cultivation Program of Natural Science Foundation of Guangdong Province (2017A03038009), National Key R&D Program of China (2018YFA0902800), Shenzhen Basic Science Research (JCYJ20190809164617205), Sanming Project of Medicine in Shenzhen PI,SAHSYSU (SZSM202011011), and Research start-up fund of part-time PI,SAHSYSU (ZSQYJZPI202003).

## Conflict of Interest

The authors declare that the research was conducted in the absence of any commercial or financial relationships that could be construed as a potential conflict of interest.
