# Supplementary material for: Revealing Prognostic Value of Skeletal-Related Parameters in Metastatic Castration-Resistant Prostate Cancer on Overall Survival: A Systematic Review and Meta-Analysis of Randomized Controlled Trial
Source: Front Oncol. 2020 Nov 19;10:586192. doi: 10.3389/fonc.2020.586192 (PMC7710983; doi:10.3389/fonc.2020.586192)
Supplement: Supplementary file 1 [file DataSheet_1.pdf]

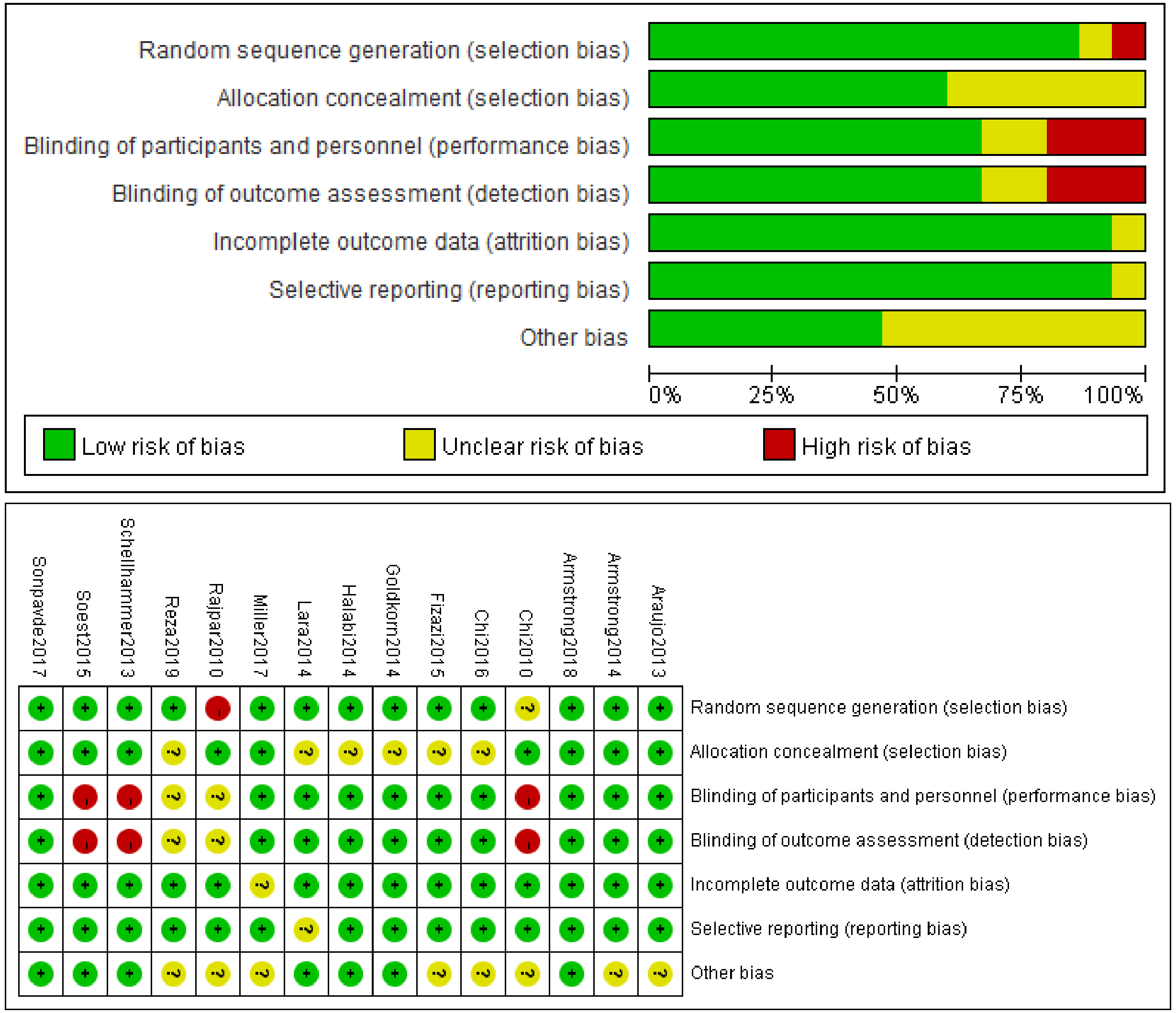

Figure S1 Summary of risk of bias assessment

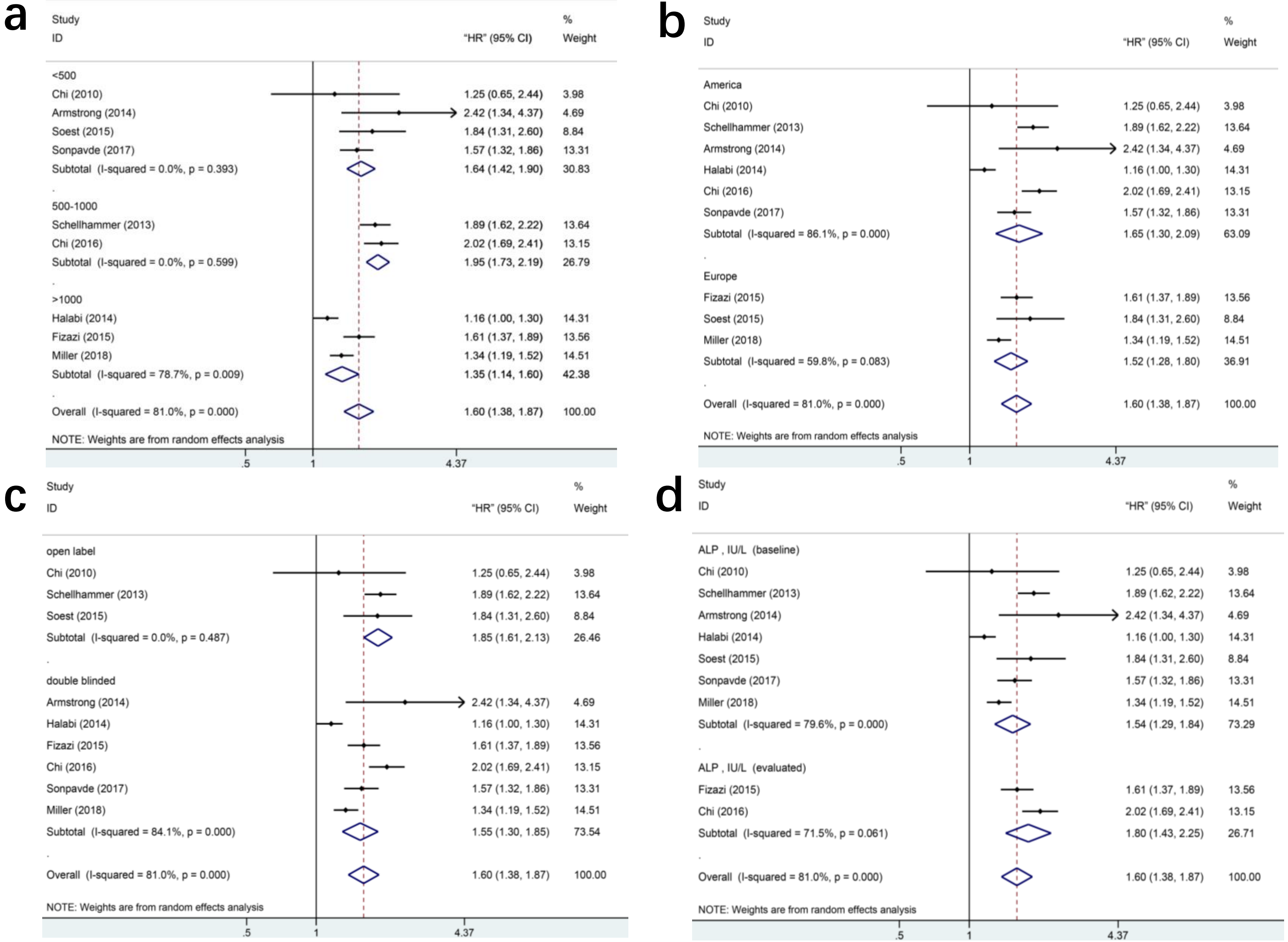

Figure S2 Forest plots of subgroup analysis of pooled ALP for OS in patients with mPCa. a sample size, b region, c study design, d serum level.

Abbreviation: HR hazard ratio; CI confidence interval; ALP alkaline phosphatase; OS overall survival; mPCa metastatic prostate cancer;

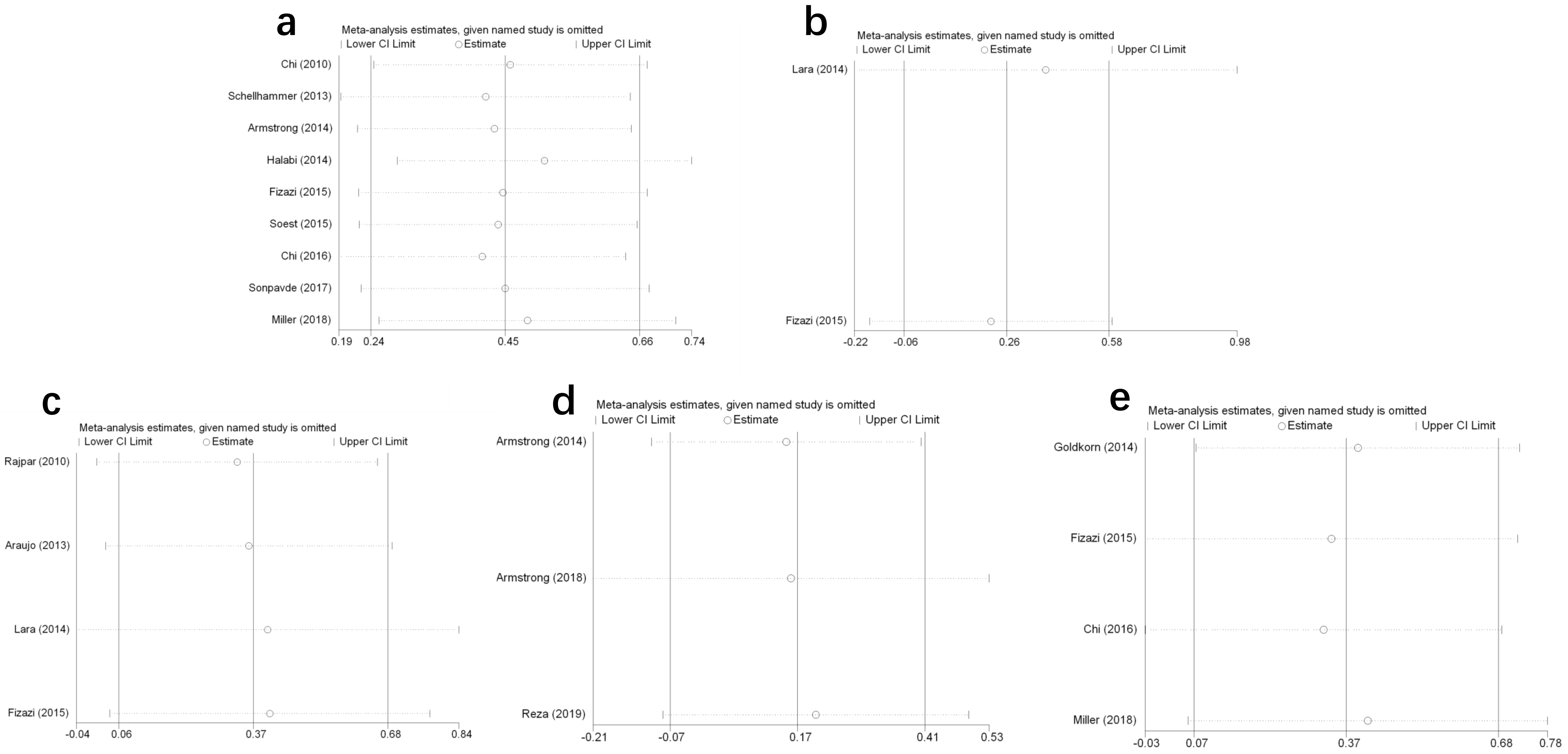

Figure S3 Sensitivity analysis of pooled ALP, BSAP, uNTx, BSI, BPI-SF score for OS in patients with mPCa. a ALP, b BSAP, c uNTx, d BSI, e BPI-SF score

Abbreviation: OS overall survival; mPCa metastatic prostate cancer; ALP alkaline phosphatase; BSAP bone-specific alkaline phosphatase; uNTx urinary N-telopeptide; BSI Bone Scan Index; BPI-SF Brief Pain Inventory-Short Form score

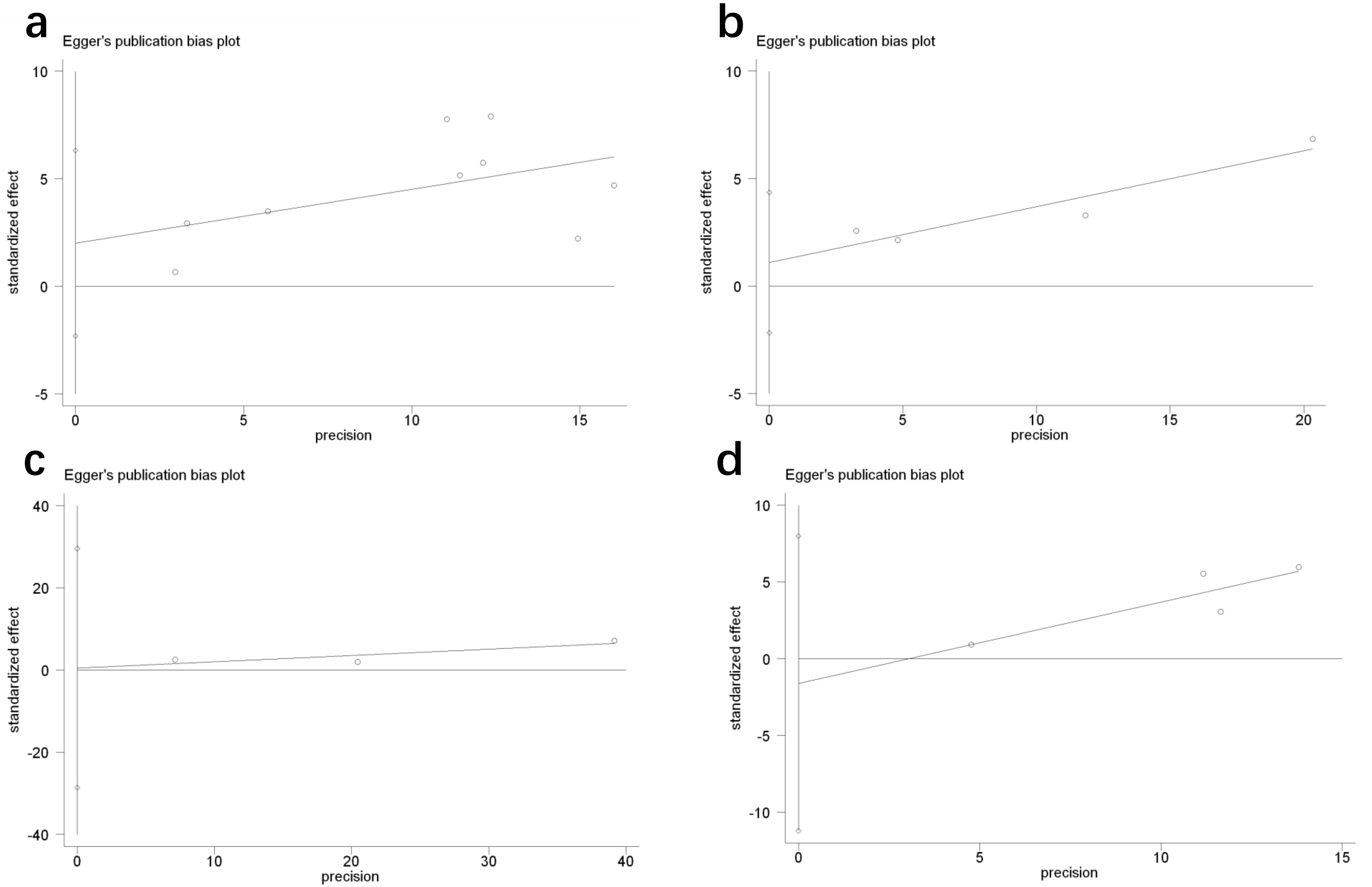

Figure S4 Egger's test of pooled ALP, uNTx, BSI, BPI-SF score for OS in patients with mPCa. a ALP, b uNTx, c BSI, d BPI-SF score

Abbreviation: OS overall survival; mPCa metastatic prostate cancer; ALP alkaline phosphatase; uNTx urinary N-telopeptide; BSI Bone Scan Index; BPI-SF Brief Pain Inventory-Short Form score

Table S1 Baseline characteristics of included studies about ALP and BSAP

Study/publish year	mean deta(range)(IU/L)	comparison	sample size	Mode of analysis	HR	95% CI	
						ll	ul
Chi2010	test: 135(54-880)<br>control: 134(47-1988)	ALP , IU/L (baseline)	82	Multivariable	1.25	0.65	2.44
Schellhammer2013	NA	ALP , IU/L (baseline)	512	Univariate	1.895	1.617	2.221
Armstrong2014	NA	ALP , IU/L (baseline)	210	Univariate	2.42	1.34	4.37
Halabi2014	test: 117<br>control: 121	ALP , IU/L (baseline)	1050	Multivariable	1.16	1	1.3
Fizazi2015	143.0 (33, 4317)	ALP , IU/L (> 143 vs. ≤ 143)	1901	Multivariable	1.606	1.367	1.889
Soest2015	125	ALP , IU/L (baseline)	114	Univariate	1.84	1.31	2.6
Chi2016	test: 134(33–4896)<br>control: 134(20–4617)	ALP , IU/L (> 160 vs. ≤ 160)	762	Univariate	2.02	1.69	2.41
Sonpavde2017	118 (27, 8560)	ALP , IU/L (baseline)	365	Multivariable	1.57	1.32	1.86
Miller2017	test: 72(67-75)<br>control: 72(65-85)	ALP , IU/L (baseline)	1088	Multivariable	1.34	1.19	1.52
Lara2014	test: 69 (41–92)<br>control: 69 (43–88)	BSAP , u/L (baseline)	788	Univariate	1.23	1.14	1.32
Fizazi2015	71 (38, 93)	BSAP , U/L (> 146 vs. ≤ 146)	1901	Multivariate	1.46	1.22	1.76

Abbreviation: ALP, alkaline phosphatase; NA; not available; HR: hazard ratio; CI: confidence interval; ll: lower limit; ul: upper limit.

Table S2 Baseline characteristics of included studies about uNTx

Study/publish year	mean deta(range) (nmol/mM CR)	comparison	sample size	Mode of analysis	HR	95% CI	
						ll	ul
Rajpar2010	19 (3–489)	uNTx , nmol/mM CR (≥20 vs.<20)	94	Multivariate	2.2	1.2	4
Araujo2013	NA	uNTx , nmol/mM CR (abnormal)	1522	Univariate	1.56	1.04	2.35
Lara2014	NA	uNTx , nmol/mM CR (baseline)	788	Univariate	1.4	1.27	1.54
Fizazi2015	51.9(4-3904)	uNTx , nmol/mM CR (>50 vs. ≤50)	1901	Multivariate	1.32	1.12	1.56

Abbreviation: uNTx, urinary N-telopeptide; NA; not available; HR: hazard ratio; CI: confidence interval; ll: lower limit; ul: upper limit.

Table S3 Baseline characteristics of included studies about BSI

Study/publish year	comparison	sample size	Mode of analysis	HR	95% CI	
					ll	ul
Armstrong2014	BSI, Bone scan progression	210	Univariate	1.42	1.08	1.87
Armstrong2018	BSI, Bone scan progression	1245	Univariate	1.2	1.14	1.26
Reza2019	BSI, Bone scan progression	1433	Multivariate	1.1	1.008	1.221

Abbreviation: BSI, Bone Scan Index; NA: not available; HR: hazard ratio; CI: confidence interval; ll: lower limit; ul: upper limit.

Table S4 Baseline characteristics of included studies about BPI-SF

Study/publish year	no. of patients	comparison	sample size	Mode of analysis	HR	95% CI	
						ll	ul
Goldkorn	92(≥4)<br>120(<4)	BPI-SF score (≥4)	212	Univariate	1.21	0.8	1.82
Fizazi2015	732(>4)<br>1169(≤4)	BPI-SF score (＞4 vs. ≤4)	1901	Multivariable	1.54	1.34	1.78
Chi2016	NA	BPI-SF score (＞4 vs. ≤4)	762	Univariate	1.64	1.38	1.96
Miller2017	66(0-1)	BPI-SF score (2,3 vs. 0,1)	1088	Univariate	1.30	1.10	1.53

Abbreviation: BPI-SF: Brief Pain Inventory-Short Form score; NA: not available; HR: hazard ratio; CI: confidence interval; ll: lower limit; ul: upper limit.
